# Ovarian dysgerminoma detected by ^18^F-FDG PET/CT technique: A case report

**DOI:** 10.1097/MD.0000000000023074

**Published:** 2020-11-06

**Authors:** Peng Wang, Yaqi Feng, Wenli Dai, Qinxue Pu

**Affiliations:** a Department of Nuclear Medicine; b Department of Pathology, Yichang Central People's Hospital, China Three Gorges University, Yichang, PR China.

**Keywords:** ^18^F-FDG, ovarian dysgerminoma, PET/CT

## Abstract

**Introduction:**

Ovarian dysgerminoma (OD) mostly affect young women, have a rapid growth rate, and could result in complications such as rupture, hemoperitoneum or torsion, and acute abdomen. However, there have been no reports of OD on ^18^F-FDG PET/CT imaging.

**Patient concerns:**

A 21-year-old female patient was admitted to our hospital on February 6, 2016, due to “reduced menstrual flow with abdominal distension for 3 months”.

**Diagnosis:**

Color Doppler ultrasound showed a large solid mass in the abdomen and pelvis. Serum carbohydrate antigen 125 (CA125) was elevated significantly. Subsequent computed tomography (CT) of chest showed a large effusion in the right thoracic cavity. Abdominal CT scan revealed the presence of a solid mass occupying a large space in the middle and lower abdomen, suggesting that it derived from the left ovary. Then, she underwent ^18^F-fluoro-2-deoxy-D-glucose (^18^F-FDG) positron emission tomography (PET)/CT examination for further diagnosis and staging. PET/CT showed a large occupying lesion in the abdomen. The maximum standardized uptake (SUV_max_) of ^18^F-FDG was 15.8. No obvious hypermetabolic metastases were observed in the other parts of the body. Postoperative pathology and immunohistochemistry confirmed the ovarian dysgerminoma.

**Interventions:**

The patient underwent surgery. Chemotherapy was successfully carried out post-operation.

**Outcomes:**

Fortunately, the patient is responding well to treatment and the postoperative recurrence-free survival time has been more than 3 years.

**Conclusion:**

OD usually occurs in young women and is characterized by large solid pelvic mass. The ^18^F-FDG PET/CT scan shows abnormally increased metabolism of the tumor. Because of the high metabolic characteristics, ^18^F-FDG PET/CT may be of great significance in the diagnosis and staging of OD.

## 1 Introduction

Ovarian dysgerminoma (OD), the most frequently encountered germ cell tumors, approximately account for 30% to 40% of the ovarian germ cell tumors and 1% to 3% of all ovarian malignant carcinoma cases.^[1–3]^ OD commonly occurs in young women, 90% of whom are younger than 30 years old (average age of 16–20 years old), and about 20% to 30% of patients are diagnosed during pregnancy.^[4–6]^ OD has a rapid growth, and could result in complications such as rupture, hemoperitoneum or torsion, and an acute abdomen.^[7]^ Here, we report a patient with OD who was diagnosed by ^18^F-fluoro-2-deoxy-D-glucose-positron emission tomography/computed tomography (^18^F-FDG PET/CT) in China.

## 2 Case presentation

A 21-year-old Chinese female patient was admitted to our hospital on February 6, 2016 due to “reduced menstrual flow with abdominal distension for 3 months”. She was previously in satisfactory health condition and had a normal menstrual cycle. In October 2015, her menstrual flow was significantly reduced, appearing dark red, but without discomfort (e.g., dysmenorrhea). There was no menstruation in the subsequent 2 months, and she experienced abdominal distension, which was obvious after eating. On January 10, 2016, the patients menstrual occurred again; however, the amount was still less than normal, with no medicine or special treatment taken during this period. The patient had no history of sexual intercourse and did not undergo gynecological examination. Her father had a history of seminoma.

On February 6, 2016, color Doppler ultrasound examination in the outpatient of our hospital showed a huge solid mass in the abdomen and pelvis, with a dimension of about 22 cm × 20 cm. It extended from the pelvic cavity to about 2 cm below the xiphoid process. The mass had an irregular shape, a clear boundary, and uneven inner echo, and the ultrasonography suggested that it might originate from ovary or retroperitoneum. The patient was hospitalized on the same day to undergo different medical examinations. On abdominal physical examination, a palpable large, fixed, solid mass in the right abdomen was observed. Anal examination revealed a mass in the pelvic and abdominal cavity with clear lower margin and no abnormalities in Douglas cavity. The results of laboratory tests that day were as follows: carbohydrate antigen 125 (CA125), 1421.0 U/ml (reference range, 0–35 U/ml); CA153, 29.5 U/ml (reference range, 0–25 U/ml); CA199, 11.9 U/ml (reference range, 0–39 U/ml); alpha fetoprotein (AFP), 1.8 ng/ml (reference range, 0–13.6 ng/ml); carcinoembryonic antigen (CEA), 1.5 ng/ml (reference range, 0–10 ng/ml). Liver function parameters were as follows: alanine aminotransferase (ALT), 43 U/L (reference range, 7–40 U/L); aspartate aminotransferase (AST), 114 U/L (reference range, 13–35 U/L). Renal function, human chorionic gonadotropin (HCG), sexual hormones, and other indicators were normal.

**Figure 1:**
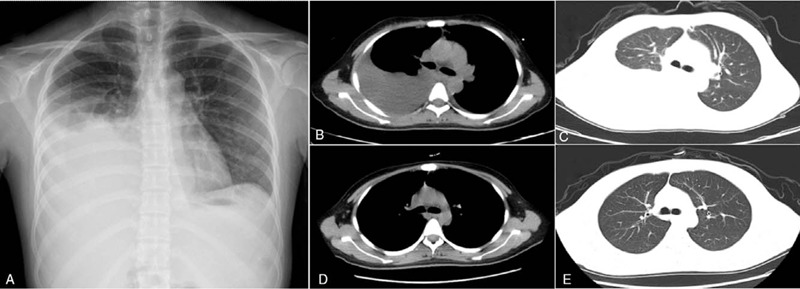
Preoperative and postoperative X-ray and CT findings. Preoperative X-ray of chest showed a massive effusion in the right thoracic cavity (A). Preoperative CT of chest showed a massive effusion in the right thoracic cavity combined with segmental consolidation of the right lower lung (B and C). CT of chest at 5 weeks after surgery showed no abnormal density in either lung or the mediastinum (D and E).

**Figure 2:**
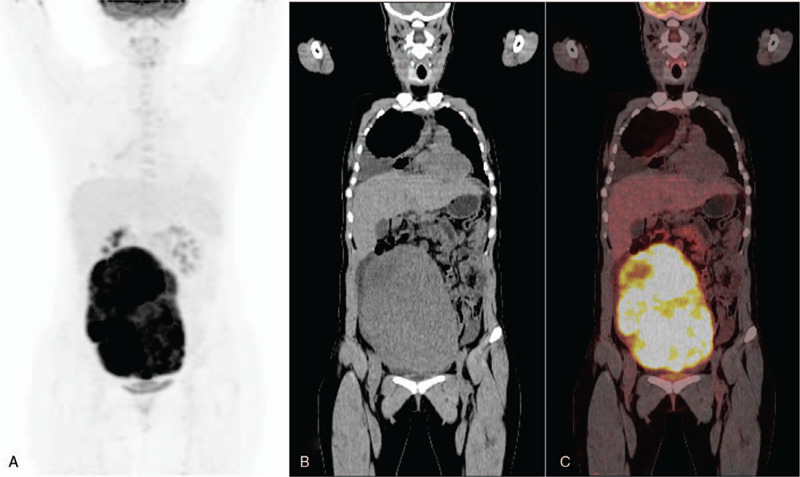
Preoperative PET/CT findings, Positron emission tomography (PET) with maximum grayscale projection (A). Coronal CT image showed a large mass in the abdominal cavity, with an uneven density, and a clear boundary (B). PET/CT fusion image showed uneven abnormal increase of tumor metabolism, with no abnormal hypermetabolic lesions in other sites (C).

Chest X-ray on the day of hospitalization showed a large effusion in the right thoracic cavity (Fig. 1A). Chest computed tomography (CT) scan showed a large effusion in the right thoracic cavity with segmental consolidation of the right lower lung (Fig. 1B and C). Abdominal CT scan revealed the presence of a solid mass occupying a large space in the middle and lower abdomen, suggesting that it originated from the left ovary. Gastroscopy revealed no obvious abnormalities in the gastric mucosa, and no biopsy was performed. PET/CT showed a large space occupying lesion in the abdominal cavity (size of approximately 15.3 cm × 10.5 cm × 23.4 cm), with uneven density and a clear boundary. The metabolism of the mass showed uneven abnormal enhancement. The maximum standardized uptake (SUV_max_) of ^18^F-FDG was 15.8. No abnormal hypermetabolic lesion was found in other sites. It was considered a neoplastic lesion derived from the left ovary (Fig. 2A, B and C).

**Figure 3:**
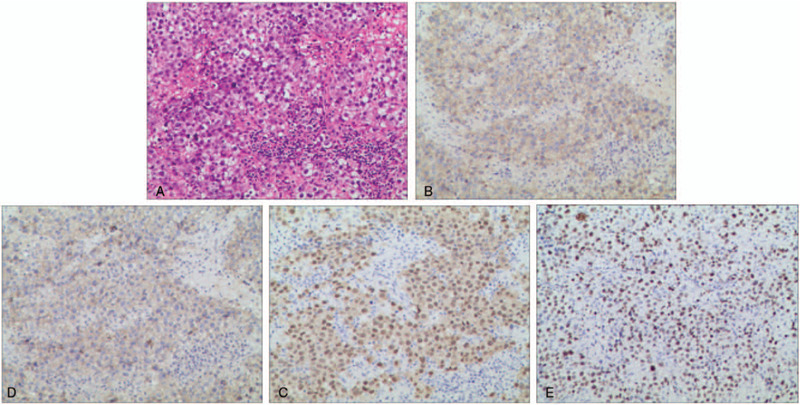
Histopathological results of the ovarian dysgerminoma and immunohistochemical staining. After hematoxylin and eosin (H&E) staining, microscopic observation revealed that tumor cells were arranged in a nested shape, regular, round and quasi-circular with size uniformity. The cells had reduced cytoplasm, clear nuclear membrane, visible nucleolus and coarse chromatin, and karyokinesis was commonly observed. Necrosis could be observed in some areas, with fine fiber separation between the tumor nests, some focal lymphocytes infiltrated between the tumor cell mass and the interstitial space, and no adenoid and papillary regions(A). Immunohistochemistry revealed CD117, PLAP, OCT3/4 were all positive, and Ki-67 (about 90% +) (B, C, D and E respectively). Other indicators such as Vim, S100, CD30, AFP, inhibin-α, CD99, CR (calretinin), and EMA were all negative.

The night following admission, the patient underwent emergency laparotomy, with left accessory resection, partial omentectomy, right ovarian biopsy, and rectouterine fossa biopsy. Intraoperative exploration showed a solid tumor of about 20 cm × 15 cm × 10 cm in the left ovary with a smooth surface. The mass was immediately sent to the department of pathology for intraoperative rapid biopsy, which revealed ovarian cancer. Examination of liver surface, omentum majus, intestinal surface, uterus, and right adnexa showed no abnormalities. Postoperative pathology revealed tumor cells arranged in a nested shape, regular, round and quasi-circular with uniform size. Necrosis could be observed in some areas, with fine fiber separation between the tumor nests, some focal lymphocytes infiltrated between the tumor cell mass and the interstitial space, and no adenoid and papillary regions (Fig. 3A). Immunohistochemistry revealed CD117, PLAP, OCT3/4 were all positive, and Ki-67 (about 90% +) (Fig. 3 B, C, D and E respectively). Other indicators such as Vim, S100, CD30, AFP, inhibin-α, CD99, CR (calretinin), and EMA were all negative. Based on these findings, the lesion was diagnosed as OD, and no tumor invasion was observed in the left fallopian tube. The left ovarian suspensory ligament stump and omentum majus were examined, and no lesions were observed. The postoperative stage was IC according to the International Federation of Gynecology and Obstetrics criteria (FIGO).

No cancer cells were found in the hydrothorax (February 14, 2016) and ascites (February 6, 2016). Three cycles of bleomycin, etoposide and platinum (PEB) chemotherapy (etoposide 725 mg + cisplatin 143 mg + bleomycin 30 mg) were administered after surgery, and no radiotherapy was carried out.

CA125 was gradually decreased after surgery: 1 week, 944.7 U/ml (elevated); 5 weeks, 51.4 U/ml (elevated); 8 weeks, 30.8 U/ml (normal). During reexamination at 5th week after surgery, the color Doppler ultrasound of the abdomen showed no abnormalities in the uterus and adnexa uteri. Besides, CT scan of chest showed no abnormalities in both lungs and the mediastinum (Fig. 1D, E). At the last follow-up on April 8, 2018, there was no sign of cancer in the abdominopelvic cavity. In addition, CA125 (22.9 U/ml), CEA (2.4 ng/ml), AFP (0.9 ng/ml), ALT (9 U/L) and AST (15 U/L) were all normal. To date, the patient has been in good physical condition without obvious discomfort. The postoperative recurrence-free survival (RFS) time has reached 36 months.

Informed written consent was obtained from the patient for publication of this case report and accompanying images.

## 3 Discussion

The majority of OD is very large (15–20 cm), which may be combined with elevated blood levels of CA125, CA19-9, AFP, and HCG. The definite diagnosis still depends on pathological examination. Positive immunohistochemical staining of CD117 is a typical marker of OD.^[8]^ OD is a moderate and low-grade malignant tumor, and the prognosis is generally satisfactory after surgery and adjuvant chemotherapy or radiotherapy.^[9]^ The patient presented here showed a large solid pelvic mass, significant serum CA125 elevation, and positive immunohistochemical staining of CD117, which is consistent with the diagnosis of OD. Postoperative CA125 progressively declined and returned to normal level within 2 months post-operation. The patient had a relapse-free survival of more than 3 years.

No previous study reported the diagnosis of dysgerminoma by PET; therefore, it is characteristic manifestations on PET have remained largely elusive. The present case showed abnormally increased mass metabolism and localized areas of reduced metabolism, which may be associated with local tumor necrosis. OD is often characterized by quasi-circular, elliptical, irregular or lobulated masses on CT. It is mainly composed of solid components, which may also be associated with cystic changes, necrosis, and calcification. The CT value of the solid component in plain scan is about 28 to 45 HU; meanwhile, the CT value in enhanced-CT scan is increased by 15 to 38 HU with the unenhanced cystic part, and about 40% of patients showed enhanced tortuous vascular shadows in the tumors.^[5]^

OD should be differentiated from the following diseases, such as hysteromyoma, epithelial ovarian cancer, endodermal sinus tumors, ovarian metastases, etc. For example, hysteromyoma rarely grow to such a large size, and FDG metabolism is low, which is usually easy to identify. Ovarian cancer is more frequent in middle-aged and elderly women, mostly presenting with cystic-solid masses, as well as being prone to metastasis, while OD is more common in young women, and manifests as a solid mass, and metastasis is rarely observed. Endodermal sinus tumors have a high degree of malignancy, rapid growth, poor prognosis, and metastatic signs at early stage, which can be accompanied by significantly increased serum AFP levels.^[10]^ Additionally, the primary lesions of most ovarian metastases can be detected by PET/CT. Therefore, ^18^F-FDG PET/CT is a promising technique for timely detection of OD, which may guide treatment as well.

## Author contributions

**Data curation:** peng wang, Yaqi Feng, Qinxue Pu.

**Project administration:** peng wang, Yaqi Feng.

**Supervision:** Wenli Dai.

**Writing – original draft:** peng wang, Yaqi Feng.

**Writing – review & editing:** peng wang, Yaqi Feng.
